# The Perfidious Experiences of Men as Palliative Caregivers of People Living with HIV/AIDS and other Terminal illnesses in Botswana. Eclectic Data Sources

**DOI:** 10.4103/0973-1075.73648

**Published:** 2010

**Authors:** Simon Kangethe

**Affiliations:** Department of Bachelor of Business Administration (BBA), Centre for Continuous Education (CCE), University of Botswana, Botswana

**Keywords:** HIV/AIDS, Men palliative caregivers, Partriarchy, Cultures, Male involvement

## Abstract

**Aim:**

The aim and objective of this scientific research article is to explore the literature with intent to raise attention to the perfidiousness of the experiences of men as palliative caregivers of people living with HIV/AIDS and other terminal illnesses.

**Methods:**

The article has utilized eclectic data sources in Botswana and elsewhere.

**Results:**

The findings indicate that care giving position of men has been found beset by: retrogressive gender unfriendly cultures; patriarchy; weaker gender empowerment campaigns; and inadequate male involvement in care.

**Conclusions:**

The article recommends: (1) a paradigm shift of structural gender dynamics; (2) making AIDS care programmes both gender sensitive and gender neutral; (3) Strengthening gender mainstreaming; (4) diluting cultures and patriarchy; (5) and signing and domesticating SADC gender protocol and other gender friendly international agreements by Botswana government.

## PROBLEM STATEMENT

Societies the world over, especially in developing world have been driven desperate by the burgeoning cases of HIV/AIDS. African South of Sahara countries that only takes a tenth of the world population continue to take the lion’s share of about 80% the global HIV/AIDS cases[1,2] This means that communities and governments have been overwhelmed in handling the HIV/AIDS load. It is in this light and context that governments have been obliged to ask its people through the community structures to extend a helping hand in order to mitigate the impact. In Botswana, this task has been achieved perfectly well with the government institutionalizing the community home based care programmes to operate within the mainstream health care systems.[3–6] However, even with this community goodwill, skewed gender phenomenon is glaring, with women largely volunteering to shoulder care and support to the people living with HIV/AIDS more than men. This has been worrying, making it difficult to take stock of the country’s success to Millennium Development Goal number three of the envisaged global gender equality, and eradicating gender retrogressive attitudes and stereotypes towards the role and place of women in the societies.[7,8] Ways, methodologies, strategies and interventions to change the current status quo of inadequate male involvement in care giving process remain a glaring lacuna that needs to be addressed.[9] Advocacy forums like this article is a welcome gesture in lobbying for a paradigm shift of having men to adequately co participate in care giving with women.[9]

## INTRODUCTION AND BACKGROUND

The role and place of men in gender development of individual countries and the world at large is critical. In this author’s opinion, their full involvement in care giving tasks will turn around the care giving environment and offload the heavy care caseload on the shoulders of women. In Sub Saharan Africa, almost 90% of AIDS care takes place at home.[10] Gender role demarcation, differentiation and socialization have skewedly allotted the task of care to women, and the girl children. This could explain why in many countries especially of the developing world, home based care is predominantly provided by girls and women.[9,11,12] Taking cognizance of the ever burgeoning cases of HIV/AIDS and therefore increased commensurate care load, this, to women, presents an arduous uphill task and gender exploitation that impedes women gender development and emancipation.[13–15] In Vietnam, for instance, 75% of all the care giving for persons living with HIV/AIDS is done by women and girls.[15] The care giving dilemma and challenge are exacerbated by the fact that most households especially in Sub Saharan Africa are increasingly becoming female headed. This has implications in terms of the economic assistance to PLWHA. In South Africa, for example, 34% of households are female headed.[15] An unfortunate state of affairs is presented when care giving has to be offered by the minors[9,12]

Psychologists have indicated the dangers of minors taking on care giving tasks as their social, psychological, physiological, and emotional capacity isnot well established, leaving chances that their growth equilibrium may be affected and may experience social challenges in their future such as lack of confidence, assertiveness and ambition.[16,17] There is, therefore, a strong and inevitable urgency for a paradigm shift to bring on board men to co participate in care giving.[6]

Ironically, and encouragingly, cases of men’s involvement in care, though on a small scale globally, regionally, nationally, and locally are however gaining momentum. Although according to a 2006 research finding in Botswana in care giving,[6] men are not doing well as caregivers, there are slight positive response as AIDS phenomenon gathers momentum immensely, challenging households, communities and governments generally. It is an incontrovertible fact that men are gradually and slowly succumbing to giving away their long held stereotypes and traditional attitudes on role demarcation and gender differentiation.[9] Men, especially those who are infected themselves, or who have worked with AIDS organizations and are knowledgeable and skilled are increasingly willing to take on physical care of sick partners and family members.[18] United Nations based research studies confirm that in Soweto, South Africa, for instance, some men have looked after sick partners or relatives effectively, but some have abandoned infected wives or thrown them out of their homes. In KwaZulu/Natal, South Africa, for example, it is not unusual to find men as primary caregivers, but most of the time they get tired of the job and abandon the practice. More often, they call their mothers or their sisters from the farms when things get bad to do care giving on their behalf.[18] More often than not, men deliver low quality care because they are not well committed destining women to be predominant caregivers.

Examples from Britain’s 1985 General Household Survey indicates that there were 3.5 million female and 2.5 male caregivers, but with women being far more likely than men to be full time caregivers.[19] A survey carried out in Spain in 1996 found that 33% of older women relied on their husbands as their caregivers.[20] This author believes that participation of men in care giving is pivotal in demonstrating and illustrating to the world how countries are progressing towards the Millennium Development Goal number three of achieving global gender equality and empowerment.[7] Besides their economic might compared to women, men are also usually endowed with muscular capacities to effect some aspects of care giving such as lifting bedridden patients in and out of beds.[18] This article, therefore, is a good advocacy forum to expedite the male involvement in care giving tempo. In Botswana, the tangible and meaningful gender development quotient needs to show that Batswana have eliminated or eradicated negative social attitudes towards the status and role of women and free them from all forms of gender based exploitations such as apparent in care giving.[7] This would be in line with some of the gender based international goals such as CEDAW (conventions on eliminations of all forms of discrimination against women)[7,8,21,22]

## FACTORS BESETTING MALE INVOLVEMENT IN CAREGIVING

### Culture

Kang’ethe[9] indicates that culture is the mirror of the society, representing the society’s thinking, its cherished values, its do’s and don’ts, and sets the pace for change in any society. It also constitutes an accumulation of values, norms, beliefs, ideologies and stereotypes that are passed from generation to generation. Different societies, therefore, have different cultural and gender packages facilitating the construction of gender reality.[9] It is an incontrovertible stark reality fact that constructing reality of a social phenomenon using culture is in itself a critical and robust natural phenomenon. In most societies of the world, socio-cultural arrangements have allotted menial tasks such as taking care of the sick to women, while men in many cultures have been assigned tasks that require more physical power input such as road building, log cutting etc. It is only the emergency of HIV/AIDS that is challenging these social structural arrangements to have men on board and co participate with women on issues pertaining to care.[9] However, the change quotient is taking too long to yield appreciable results. Dismantling or rearranging cultural strong bonds has to take long as the bond formation also took long. This explains why many men in many societies still lag behind accepting their new forced obligation[22]

### Patriarchy

Patriarchy and culture are known to influence and strengthen one another. Patriarchy is traditional customs and beliefs that gives men domination over women[23] Since gender power relationship between men and women has been skewed in many societies of the world, with men wielding immense powers, and sometimes using this power to oppress and suppress women and children, many people associate patriarchy with male dominance.[23] Patriarchy has also found strength and support from many religious doctrines and principles that see the woman as subordinate to man other than being a complement.[9,12,23] This results in men allocating and giving orders to their female counterparts. The current national campaigns and advocacy for men to increase their involvement in issues of health care have not found a good and strong platform to achieve the desired results. Men are still operating behind the patriarchal curtains or lenses to shun or justify their non performance in terms of care giving.[6,9,23]

### Weaker gender empowerment campaigns

Though gender based bodies are increasing their momentum to lobby, advocate for gendered approach to development and life perspectives, these bodies in Botswana are very few compared to the need and urgency to have adequately mainstreamed gender by the year 2015 in order for Botswana to take stock of its Millennium Development Goals achievement, and also to fulfill its national goals enshrined in its Vision 2016 document.[7,24] Though Botswana lacks sufficient number of non governmental organizations generally,[25] Emang Basadi NGO and Women affairs Department have made robust and gigantic steps in educating a bigger part of the society, especially the urban based, but the larger majority of the people living in the rural areas are not benefiting from this gender education.[26] This is a challenge which the government of Botswana is known to be facing head on. Weaker gender campaign directly or indirectly impacts on care business. This is because if gender is well mainstreamed, societies are likely to throw away some cultural and stereotypical ideologies that encourage gender skewed perspectives towards health issues such as care giving. This is evidenced by the fact that in most developed countries with a health gender balance, care can increasingly be done by men as well as women without much fuss.[22]

General communities in Botswana have not seen the need to empower and propel women into the political realm. This is explained by the fact that only a few women have been elected to go to parliament since independence.[27] This is despite the fact that women in many spheres of life in Botswana have proved to be very competitive. They have immensely challenged men in that most middle level managers in Botswana are increasingly becoming women. This could explain the fact that women’s efforts especially in education have contributed to the country being a shining example in Africa, scoring the highest Gender Development Index (GDI) in Africa.[22] Ironically, women display marginal political performance that need to be addressed. This is why the SADC Gender protocol document is calling for a 30% women representation in both parliamentary and civic seats in the Southern African region[27] This may explain the power quotients and thinking surrounding gender empowerment and dispensation in Botswana. This should be influencing the decision making processes as to how each gender sees the challenge of sharing care giving, for example. However, the country’s political goodwill to gender development is immensely on the increase.[27,28]

### Empirical inadequate male involvement in care

Experience of men in care giving of PLWHA in Botswana has not been encouraging. Several researches have indicated preponderance of women in the care field. This is a big blow leading to the counting of losses as far as achieving the Millennium Development Goals especially of gender equality is concerned. It also has an impact towards achieving the country’s Vision 2016 goals especially in becoming an AIDS free generation.[7,24] A study on care giving carried out in Botswana by Munodawafa[5] had all females except one in Tutume, while in Molepolole all caregivers were females. Studies in Botswana by Atta and Fidzani[11] had similar findings that over 50 % of caregivers in most of the Botswana CHBC programmes are elderly women who may not adequately stand care giving demands due to their advancing age. According to McDonnel, Brennam, Burnham and Tarantola,[29] the traditional safety nets providing for care giving could be stretched enormously beyond its capacity with the resultant inadequate productivity if the care giving is to be left only to the elderly. This indicates that the campaign and advocacy for diluting gender rigidities and complexes to change the strongly held gender demarcation and role differentiation need to be strengthened. Gender mainstreaming to all the social institutions needs to be strengthened. According to feminists, such as Kelesetse,[14] leaving women to perform care duties alone is gender exploitation, a human rights abuse; and also contributes to feminization of poverty.[22,26]

Studies in Western world indicate a different gender attitude to care giving.[19] In United States, for instance, older women and men are providing care to their grandchildren. This ranges from occasional babysitting to being custodial grandparents. In 1997, statistics in United States indicated that 3.9 million children (5.5% of all the children by then under the age of 18) lived in a household maintained by their grandparents (both men and women). Since 1990, the number of children in US living in households headed by grandparents has greatly increased. This could be attributed to factors such as divorce, HIV/AIDS, and drug abuse.[30]

## ROBUST INTERVENTIONS TO INCREASE MEN INVOLVEMENT IN CARE GIVING

### Effecting paradigm shift of structural gender dynamics

Due to huge gender disparities that inform gender role demarcation and differentiation, the role of care giving in many developing countries such as Botswana has widely and largely been shouldered by women and the girls.[9] The magnitude of care and care giving that HIV/AIDS has imposed on the communities calls for a paradigm shift that will dilute these gender dynamics allowing men to adequately participate in care giving together with women and the girls.[15]

Men with cultural and patriarchal ordained leadership powers that give them leeway to control much of the world in which women live must be persuaded to be partners of social change. Programs targeting women must adequately learn to embrace men as partners in order to nurture social structures that are more supportive to women. Men’s participation in home based care and other support programs would be one way of heeding their responsibility for the health and welfare of their communities and societies in general.[9,15] This discussion paper propels men as well as boys to answer the challenge of men-women cooperation in care giving and discard all the harmful stereotypes of masculinity to confront the scourge of HIV/AIDS in their communities.[15]

### Make AIDS care programmes both gender sensitive and gender neutral

In Botswana, the issue of male involvement in health care programmes poses immense challenge as men’s participation and involvement records show dismal results. In Kang‘ethe’s 2004 qualitative informal investigation of Kanye CHBC, he found only one male volunteer among the 83 community volunteers.[31] This is reinforced by this author’s 2005-2006 research in the same Kanye CHBC in which out of his 82 primary caregivers that he interviewed, only two were men. Policy analysis’s possible explanation of this gap suggests the challenge might be rooted and investigated from program’s formative conceptualization that overlooked the gender component. Taking a programme such as prevention of mother to child transmission (PMTCT) in Botswana, there was an oversight during its formative stages, making the policy to skew towards women involvement. This is because prior policy implementation and operationalization targeted women. The antenatal cases from which national HIV/AIDS statistics in Botswana are computed have largely been drawn from women, and comprising of only a few men who go to the clinics suggesting challenges of sexually transmitted nature.[32] Due to this scenario, many HIV/AIDS programmes in Botswana have been viewed by men as well as women to be women’s business. With time, this saw men lagging behind the fray until the challenge became grave. This was also informed by the knowledge and realization that men are perfect drivers of the epidemic compared to women.[33,34] Any policy conceptualization, operationalization, and implementation need to be gender neutral, sensitive and gender focused.[9,23] It is still pertinent that policy reinforcement and implementation to woo men into involvement and contribution in care programmes is continued.

### Gender mainstreaming

Gender mainstreaming education in Botswana is still not adequate despite the country’s high Gender Development Index (GDI) compared to other countries of the developing world.[27] Gender Development Index (GDI) measures the competitiveness of women vis-a-avis their male counterparts.[22] The University of Botswana’s introduction of the politics of gender course that this author teaches is one such pivotal strategy to infuse and inculcate gender messages into the University education students. Such courses are bound to change the mindset of both men and women, and dilute gender biased strategies, stereotypes, attitudes, socialization and cultures which have been richly grounded into people’s lives and minds in centuries. It is timely recommendable that all the social institutions especially the schools be targeted for gender mainstreaming. Integrating gender lessons into the educational curriculum would be a central intervention towards gender neutrality and convince men to take the same burden of care alongside women.[8,9]

### Signing and domestication of SADC gender protocol and other gender friendly international covenants by Botswana government

Though the country has ratified several gender friendly conventions and testaments such as CEDAW (elimination of all forms of discrimination against women), and Beijing Convention leading to formulation of gender policy framework, the challenge of gender awareness, implementation and operationalization of these frameworks has taken too long to take strong roots. Botswana has not yet signed the South African Development Community (SADC) gender protocol document that address many gender gaps facing women of SADC region.[8,21,27] The government of Botswana, however, claims it has the goodwill. Ironically, It indicates it cannot sign the document because there are several contentious issues in the charter that needs to be cleared, to be subjected to adequate community consultations and national debates for document ownership purposes. The issue of signing, and domestication needs to be revisited and propelled by robust policy and governmental goodwill. Changing men’s mindset is slowly happening, but at a snail’s pace. Methodologies to adequately challenge their mindset are topical, timely and overdue, so that they can robustly understand the challenge that gender insensitivity and imbalance poses to gender and national development. While Botswana may have succeeded in giving women higher posts in the government, the issue of women representation in parliament leaves a glaring lacuna. This author believes this can strongly be addressed by signing and operationalizing the SADC gender protocol[27] This gesture could possibly increase advocacy for women empowerment that would possibly trickle down to communities at village level to convince men to handle care giving tasks equally with women. Women also need to change their mindset to welcome the new gender development and dispensation.[8,9]

### Diluting cultures and patriarchy

According to the 1995 United Nations Human Development report, efforts towards eliminating gender retrogressive cultures can bear great dividends in gender development.[22] Many cultures especially traditional ones in Africa view women as servants of men and according to Lekoko, as vessels of childbearing[23] This has made the full potential and productive capacity of woman not to be realized. According to this author, trapping women’s power and capacities impacts negatively towards the countries’ Gross National Product (GNP). Diluting some of these gender-unfriendly cultures would aid women enjoying their human rights enshrined in the global human rights conventions and agreements in which many countries of the developing world are solid signatories. Domesticating, implementing and operationalizing these international conventions and agreements appear to be taking snail’s speed.[26] It appears that although many countries signed the agreements, the people were neither widely informed, nor adequately consulted, or they have not been in a position to fully comprehend their implications. In the same vein, patriarchy or male dominance needs to be weakened. This will release and unleash the trapped energy within the women folk. This will definitely impact positively on the countries’ Gross National Product and women’s human rights.[22]

## CONCLUSIONS

Men’s position in gender development in Botswana and elsewhere is pivotal. The heavy caseload that HIV/AIDS has imposed upon communities and governments calls for a change of communities’ mindset, cultures and ideologies that have imposed care giving tasks predominantly on women. Though this has been the cultural and socialization scenario across many countries, especially of the developing world, it is no longer logical with the burgeoning cases of HIV/AIDS. Men are endowed with economic might as well as muscular prowess that can contribute positively and immensely to care giving, if they change their current status quo thinking, and come on board to co- participate in care giving with women. Governments, NGOs and private sector should increase their education, advocacy and lobbying activities to ensure that cultural and patriarchal dynamics that perpetuate gender inequality are adequately addressed, to bring in a new light of understanding and dispensation where men and women equally participate as caregivers of persons living with HIV/AIDS.
